# Gastrointestinal parasites and molecular characterisation of *Cryptosporidium* spp. and *Giardia* sp. in ruminants and other domestic animals in Corsica (France), a Mediterranean island environment

**DOI:** 10.1051/parasite/2026059

**Published:** 2026-10-29

**Authors:** Sébastien Grech-Angelini, Venceslas Villier, Jean-Marc Santini, Michèle Riera, Agnès Guillaume, Damien Costa, Abdelmounaim Mouhajir, Caroline Boulocher, Loïc Favennec, Romy Razakandrainibe

**Affiliations:** 1 ULR 7519 IDEALISS, Collège vétérinaire, Institut Polytechnique UniLaSalle Rouen France; 2 Commission Parasitologie SNGTV Paris France; 3 UR 75100 ESCAPE, Université de Rouen Normandie Rouen France; 4 Laboratoires de la Collectivité de Corse (Corse-du-Sud et Haute-Corse) Ajaccio/Bastia, Corsica France; 5 GDS Corse Ajaccio, Corsica France

**Keywords:** *Cryptosporidium* spp., *Giardia* spp., Endoparasite worms, Domestic animals, Corsica (France)

## Abstract

Corsica is a mountainous French island in the northwestern Mediterranean Sea characterized by a wide diversity of natural environments where many interactions between humans, domestic animals and wild fauna occur. Gastrointestinal parasitism is a major concern for livestock productivity. Among these parasites, *Cryptosporidium* spp. and *Giardia duodenalis* are zoonotic, cosmopolitan protozoa frequently implicated in diarrheal diseases in both young animals and children. Despite an apparent favourable epidemiological context, gastrointestinal parasites remain understudied in Corsica. Between May 2023 and May 2024, 281 faecal samples were collected: 261 pooled ruminant samples (five animals per pool) from goats (*n* = 121), sheep (*n* = 117), and cattle (*n* = 23) across 159 farms and 20 individual samples from horses (*n* = 14) and pigs (*n* = 6). Coproscopic analyses detected at least one gastrointestinal parasite in 93% of all samples. Strongyle eggs (91%) and coccidian oocysts (72%) were most prevalent in ruminants. Molecular analyses (including real-time PCR and genotyping) identified *Cryptosporidium* spp. in 11 samples (3.9%): nine goats, one sheep, and one pig. Three zoonotic species were confirmed: *Cryptosporidium parvum* (genotype family IId) in five goat samples and one sheep sample, *C. ubiquitum* in one goat sample, and *C. scrofarum* in one pig sample (first report of this species in France). *Giardia duodenalis* assemblage E was detected in a goat herd. This study documents widespread gastrointestinal parasitism in Corsican livestock. While no human cases of giardiasis or cryptosporidiosis have officially been reported in Corsica, this first detection of zoonotic species in livestock highlights potential public health risks and supports the need for evidence-based prevention strategies for humans and animals.

## Abstract (fr)

La Corse est une île montagneuse française située au nord-ouest de la mer Méditerranée, caractérisée par une grande diversité de milieux naturels où s'opèrent de nombreuses interactions entre l'homme, les animaux domestiques et la faune sauvage. Le parasitisme gastro-intestinal constitue une préoccupation majeure pour la productivité de l'élevage. Parmi ces parasites, *Cryptosporidium* spp. et *Giardia duodenalis* sont des protozoaires zoonotiques cosmopolites, fréquemment impliqués dans des maladies diarrhéiques tant chez les jeunes animaux que chez les enfants. Malgré un contexte épidémiologique apparemment favorable, les parasites gastro-intestinaux restent peu étudiés en Corse. Entre mai 2023 et mai 2024, 281 échantillons de matières fécales ont été collectés : 261 échantillons groupés de ruminants (cinq animaux par lot) provenant de chèvres (*n* = 121), de moutons (*n* = 117) et de bovins (*n* = 23) répartis dans 159 élevages, ainsi que 20 échantillons individuels provenant de chevaux (*n* = 14) et de porcs (*n* = 6). Les analyses coproscopiques ont révélé la présence d'au moins un parasite gastro-intestinal dans 93 % de l'ensemble des échantillons. Les œufs de strongles (91 %) et les oocystes de coccidies (72 %) étaient les plus fréquents chez les ruminants. Les analyses moléculaires (incluant la PCR en temps réel et le génotypage) ont identifié *Cryptosporidium* spp. dans 11 échantillons (3,9 %) : neuf chez des chèvres, un chez un mouton et un chez un porc. Trois espèces zoonotiques ont été confirmées : *Cryptosporidium parvum* (famille de génotypes IId) chez cinq chèvres et un mouton, *C. ubiquitum* dans un échantillon caprin, et *C. scrofarum* chez un porc (première signalisation de cette espèce en France). *Giardia duodenalis* (assemblage E) a été détecté dans un troupeau de chèvres. Cette étude met en évidence un parasitisme gastro-intestinal répandu au sein du cheptel corse. Bien qu'aucun cas humain de giardiose ou de cryptosporidiose n'ait été officiellement signalé en Corse, cette première détection d'espèces zoonotiques chez les animaux d'élevage souligne les risques potentiels pour la santé publique et justifie la mise en place de stratégies de prévention fondées sur des données probantes, tant pour l'homme que pour l'animal.

## Introduction

Parasitic infections, particularly those caused by gastrointestinal nematodes (GINs) and *Eimeria* spp., are the primary agents responsible for production decreases in many intensive and extensive farming systems [8]. These infections result in indirect economic losses, including reduced milk production, decreased weight gain, increased feed requirements, and veterinary treatment costs. In more severe cases, they can also lead to direct losses associated with animal mortality [8]*.* Control measures against gastrointestinal parasites primarily rely on the use of drugs. However, their widespread and uncontrolled use has led to the emergence of resistance, especially for anthelmintic drugs [39]. *Giardia* spp. and *Cryptosporidium* spp. are the most common enteric parasites of domestic animals, including livestock, dogs, and cats [4, 15, 37]. Both protozoans are common human parasites that are transmitted through contaminated drinking water or food [17, 37]. Cryptosporidiosis and giardiasis are usually self-limiting in immunocompetent individuals (animals and humans). Both are potentially severe, life-threatening diseases in immunocompromised or young individuals. They cause gastroenteritis and nutritional disorders, primarily responsible for diarrhoea. The life cycles of both *Giardia* and *Cryptosporidium* are direct, and the infective stages of both parasites (*Giardia* cysts and *Cryptosporidium* oocysts) are encysted when released in the faeces and are immediately infectious. Cysts and oocysts remain infectious for months in cool, damp areas and rapidly accumulate in environments [43]. Both are resistant to disinfectants at the concentrations and exposure times commonly used in animal husbandry and have low infectious doses [6].

Corsica is a French island in the western Mediterranean Sea, situated 15 km north of Sardinia and 90 km west of Tuscany, Italy (Fig. 1). It is the fourth largest Mediterranean island and the most mountainous and forested of these islands. Corsica consists of two administrative departments (Corse-du-Sud and Haute-Corse) and 360 municipalities, the smallest administrative unit in France (Fig. 1). Important activities in Corsica include tourism (3.3 million people annually for 330,000 permanent inhabitants), extensive or semi-extensive farming (100 000 sheep, 45 000 goats, 62 000 pigs, and 39 000 cattle), hunting, and hiking [33, 34] The proximity and frequent overlap of habitats between domestic ruminants and wild fauna, particularly in extensive grazing systems, increase the likelihood of cross-species pathogen transmission. Shared pastures, water sources, and migration corridors are common grounds for maintaining and amplifying faecal-oral and vector-borne parasitic cycles [23]. Based on current coproscopic analyses and observations from slaughterhouses, gastrointestinal parasites are widely recognized by local veterinarians and farmers as a significant health concern on Corsica Island. However, no official data or peer-reviewed publications are currently available to substantiate this concern.

**Figure 1:**
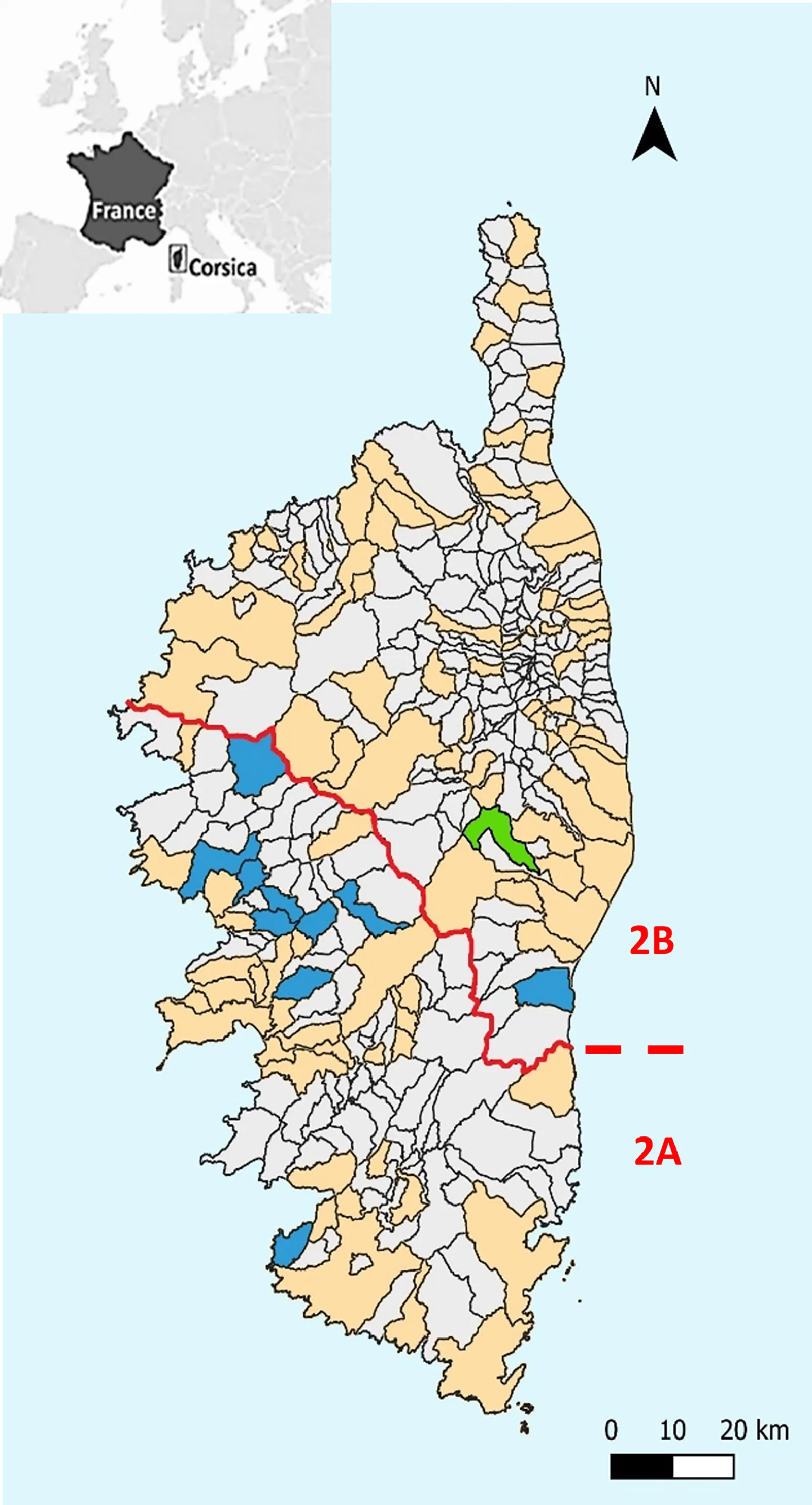
Location of Corsican municipalities where faecal samples were collected and municipalities with detected *Cryptosporidium* spp. and *Giardia* spp. Green: municipalities where *Giardia* spp. was detected; blue: municipalities where *Cryptosporidium* spp. was detected; light orange: municipalities where neither *Giardia* spp. nor *Cryptosporidium* spp. was detected; grey: municipalities not sampled. The red dotted line indicates the boundary between the two departments.

To date, the circulation of *Cryptosporidium* and *Giardia* species on the island of Corsica remains largely unknown. No human cases of giardiasis or cryptosporidiosis, nor any positive water samples for these two protozoan parasites, had ever been reported by local public health authorities. No national survey on these two parasites has included Corsica Island [11], and only scattered observations on the presence of these parasites have been documented. A recent study identified *Cryptosporidium viatorum* in rats from Corsica [19]. Local veterinarians have long recognized giardiasis as a common cause of diarrhoea in young dogs and cats; however, unlike mainland France and other parts of Europe, no epidemiological data are currently available on this parasite in Corsica [4]. The objective of this one-year study (May 2023–May 2024) was to provide an overview of the presence of gastrointestinal parasites in animals on this Mediterranean Island, with a particular emphasis on small ruminant livestock. The study also aimed to genetically characterize species and subtypes, when possible, belonging to the genera *Giardia* and *Cryptosporidium*. Such data are critical for improving livestock management practices and for assessing potential public health risks, particularly in relation to zoonotic species.

## Materials and methods

### Ethical standards

The protocol for this study did not require review or approval from the Animal Studies Ethics Committee. Written informed consent was obtained from all animal owners prior to sample collection, authorizing the anonymous use of data for epidemiological research and publication purposes. The stool collection procedure was non-invasive and ensured that no harm was caused to any of the animals involved in the study. Faecal samples were transported frozen in triple-layer packaging, in compliance with biosafety and biological material transport regulations.

### Faecal sample collection

Faecal samples were collected by owners or livestock technicians as part of a yearly parasitism monitoring programme in Corsican livestock (goats, sheep, cattle, and pigs) and horses, coordinated by a local animal health protection association (GDS Corse, *Groupement de Défense Sanitaire* of Corsica). These samples were subjected to coproscopic analysis. For ruminants, the objective of this monitoring programme was to assess the overall parasitic burden at the herd level. Ruminant samples therefore consisted of pooled faeces from five animals grouped by age category (under or over six months) and sex. Each pool included animals belonging to the same age class and sex. In contrast, pig and horse samples were collected individually, as the objective was to assess the parasitic status of specific animals.

### Coproscopy

The collected faecal samples were transported by owners or livestock technicians to the two official departmental laboratories of Corsica (located in Corse-du-Sud and Haute-Corse, Fig. 1). Coprological examinations were carried out using the standard McMaster flotation technique [24, 31]. Before microscopic analysis, the macroscopic characteristics (consistency, colour, etc.) of each sample were assessed. The detection of parasitic components, including nematode and cestode eggs, lungworm larvae, and *Eimeria* spp. oocysts, was subsequently performed according to standard parasitological diagnostic procedures. Briefly, for each sample, 3 grams of faeces, predominantly pooled samples (individual analyses were limited to domestic carnivores, horses, and pigs), were homogenised with 42 mL of saturated magnesium sulphate (MgSO_4_) solution (specific gravity = 1.26) and examined microscopically. Whenever applicable, semi-quantitative estimation of parasitic burden was carried out (Table 2). The infestation levels were expressed as eggs per gram of faeces (EPG) for gastrointestinal nematodes (GINs) and oocysts per gram (OPG) for *Eimeria* species. Due to the limited sensitivity of the McMaster technique in detecting certain parasitic stages, the identification of trematode and cestode eggs, as well as lungworm larvae, was documented solely in qualitative terms (presence or absence).

### Molecular detection and identification of *Cryptosporidium* and *Giardia* species

#### DNA extraction

The faecal samples were stored at −20 °C in the departmental laboratories of Corsica until they were shipped to the French National Reference Centre for Cryptosporidiosis, Microsporidiosis, and Other Digestive Protozoan Infections (CHU de Rouen, Normandy, France) for molecular analysis. After an initial pretreatment step, faecal DNA was extracted from 200 mg of homogenised faecal material. The pretreatment was performed using a FastPrep-24 5G system (MP Biomedicals, Irvine, CA, USA) at a speed of 6 m/s for 90 s, performed over seven cycles with a 20-second pause between each cycle. The extraction process was performed using a QIAamp PowerFecal Pro DNA Kit (QIAGEN, Hilden, Germany), following the manufacturer’s protocols, and assisted by a Qiacube Connect DNA extraction device (QIAGEN). The extracted DNA was stored at −20 °C until further analysis.

#### *Cryptosporidium* species identification and genotyping

All samples were analysed using molecular methods for species identification, and positive samples were genotyped in a subsequent step.

As described by Elwin and colleagues, the genus *Cryptosporidium* was screened using real-time PCR targeting a hypervariable region of the SSU rRNA gene [16]. The thermocycling conditions were as follows: 95 °C for 3 min, followed by 45 cycles of 95 °C for 15 s and 60 °C for 60 s. Data were collected during each 60 °C annealing/extension phase. Sequence-based characterisation of *Cryptosporidium* was performed to determine the species. *Cryptosporidium* species were detected by nested PCR amplification of the SSU rRNA gene [27]. Positive samples were then further characterised by sequencing a fragment of the gp60 gene. Primers AL3531 and AL3533 were used in the primary PCR, while primers AL3532 and LX0029 were used in the secondary PCR to amplify a fragment of approximately 364 bp [21]. Each amplification run included a negative control (PCR water) and two positive controls (genomic DNA from *C. parvum* oocysts purchased from INRAE Centre Val de Loire-Nouzilly, France, and *C. hominis* genomic DNA from a faecal specimen collected at Rouen University Hospital). The products were visualised in 2% agarose gels using ethidium bromide staining, and sequencing was carried out for species identification and subtyping. PCR amplicons were purified using exonuclease I/shrimp alkaline phosphatase (Exo-SAP-IT) (USB Corporation, Cleveland, OH, USA). They were sequenced in both directions using the same PCR primers at 3.2 μM in 10 μL reactions with Big Dye™ chemistry in an ABI 3500 sequence analyser (Applied Biosystems, Foster City, CA, USA). Raw chromatograms from forward and reverse sequencing reactions were visually inspected, and low-quality bases at the 5′ and 3′ ends were trimmed before generating consensus sequences. Consensus sequences were obtained by aligning forward and reverse reads and manually resolving ambiguous nucleotide positions. The resulting sequences were compared with reference sequences deposited in the GenBank database using the Basic Local Alignment Search Tool (BLAST) to determine the closest species or assemblage. Representative reference sequences were subsequently retrieved from GenBank and aligned with the sequences generated in this study using ClustalW/MUSCLE implemented in IQ-Tree. Phylogenetic relationships were inferred using the Maximum Likelihood method with 1 000 bootstrap replicates, and the resulting trees were used to confirm the taxonomic placement of the obtained sequences.

#### *Giardia* spp. detection and assemblage identification

Real-time PCR was conducted using iQ^TM^ Supermix (Bio-Rad Laboratories, Marnes-la-Coquette, France), targeting the small subunit (*ssu*) rRNA gene of *G. duodenalis,* specifically with primers and probes [41].

DNA samples with a positive result on *ssu*-PCR were subsequently investigated using a multilocus genotyping (MLG) scheme that amplified partial sequences of the *gdh*, *bg*, and *tpi* genes of *G*. *duodenalis*.

To amplify a ~511-bp fragment of the *gdh* gene, a semi-nested PCR was performed. The protocol included the outer primer set GDHeF (5′–TTCCGTRTYCAGTACAACTC–3′) and GDHiR (5′–ACCTCGTTCTGRCTGGCGCA–3′), and the inner primer set GDHiF (5′–ATGACYGAGCTYCAGAGGCACGT–3′) and GDHiR (5′–GTGGCGCARGGCATGATGCA–3′) [12].

For the *bg* gene, a partial sequence of ~511 bp was amplified using the outer primer pair G7 (5′–AAGCCCGACGACCTCACCCGCAGTGC–3′) and G759 (5′–GAGGCCGCCCTGGATCTTCGAGACGAC–3′) and the inner primer pair BG-F (5′–GAACGAACGAGATCGAGGTCCG–3′) and BG-R (5′–CTCGACGAGCTTCGTGTT–3′), as described previously [29]. For the *tpi* gene, a ~530-bp fragment was amplified using the outer primer pair AL3543 (5′–AAATIATGCCTGCTCGTCG–3′) and AL3546 (5′–CAAACCTTITCCGCAAACC–3′), and the inner primer pair AL3544 (5′–CCCTTCATCGGIGGTAACTT–3′) and AL3545 (5′–GTGGCCACCACICCCGTGCC–3′), as described by Sulaiman *et al.* [42].

For *Giardia duodenalis* detection, the following controls were used. During DNA extraction, a negative extraction control (molecular-grade water processed in parallel with samples) was included in each batch to monitor potential contamination. For the SSU rRNA real-time PCR, each run included a positive control consisting of DNA from the reference strain *G. duodenalis* WB clone 6 (ATCC 50803), maintained in culture at the UR7510 ESCAPE laboratory, and a negative PCR control (PCR-grade water). For nested PCR assays targeting the *gdh, bg*, and *tpi* genes, the same positive control DNA was used, together with negative controls included in both the primary and secondary reactions. All negative controls remained negative. All pre- and post-amplification steps were performed in physically separated areas using dedicated equipment to prevent cross-contamination.

All Nested PCR assays were conducted using 400 nM of each primer, 5 μL of DreamTaq buffer, 0.2 mM of dNTP, 1.25 U of DreamTaq polymerase, and 5% of DMSO (100%) was added to the PCR mix. Finally, 5 μL of DNA template was added to each reaction, and all assays were performed in duplicate.

All amplified products were sequenced on an ABI 3500 sequence analyser (Applied Biosystems) using the BigDye^®^ Terminator v3.1 cycle sequencing kit described above. Nucleotide sequence accuracy was confirmed by sequencing two separate PCR products. Assemblages and subtypes were identified by aligning nucleotide sequences with known reference *tpi, gdh*, and *bg* gene sequences of *G. duodenalis* available in GenBank using BLAST (http://www.ncbi.nlm.nih.gov/BLAST/).

### Data analysis

The rates and means were calculated with their corresponding 95% confidence intervals. Pearson’s Chi-squared test and student’s *t*-test were used to compare percentages and means, respectively. Statistical differences between groups were considered significant at *p* < 0.05 performed using Stata software (version 13, StataCorp, College Station, TX, USA). Spatial distribution maps (Fig. 1) were generated using QGIS software (version 3.4).

## Results

### Animal sample population

Between May 2023 and May 2024, 281 faecal samples were collected. Eighty-five were obtained from the Corse-du-Sud department, while 196 came from Haute-Corse (Table 1; Fig. 1). The samples were collected from 171 different owners, spread across 102 out of the 360 municipalities on the island of Corsica (Fig. 1). All samples of ruminants (n = 261) comprised pooled faecal material from five animals, representing 1 300 animals reared on 159 farms. Goats were the predominant sample group, representing 43% (*n* = 121) of the total (*n* = 281), followed by sheep at 42% (*n* = 117), and then cattle at 8% (*n* = 23) (Table 1). The samples from the ruminant population (*n* = 261) were mainly from females over 6 months of age (96%), 9 samples were collected from young female animals (< 6 months of age) and 10 from adult males. In addition, individual faecal samples (*n* = 20) were collected from 14 horses and 6 pigs (Table 1).

**Table 1:** Distribution of faecal samples by animal species and department (Corsica, May 2023–May 2024).

Samples	2A*	2B*	Male/Female	< 6 months /Adult	Corsica	Coproscopic analysis (Mc Master)	Samples positive for at least one gastrointestinal parasite
Sheep^**^	20	97	4/113	6/111	117	117	112 (96%)
Goats^**^	51	70	6/115	8/113	121	119	113 (95%)
Cattle^**^	5	18	0/23	1/22	23	23	21 (91%)
Horses	7	7	9/5	0/14	14	14	9 (64%)
Pigs	2	4	0/5	0/6	6	6	5 (83%)
Total	85	196	19/262	9/272	281	279	260 (93%)

*2A: Department of Corse-du Sud, 2B: Haute-Corse, see Figure 1.

**: Samples consisting of pools of five animals from the same farm (grouped by age and sex).

### Endoparasites found by coproscopic examination

Of the 281 faecal samples collected, 279 were analysed using the McMaster coproscopic technique. In 93% of all analysed animal samples, at least one species of endoparasite was detected (Table 1). Focusing on ruminant species, representing the three most frequently sampled host species in this study (*n* = 259 samples with coproscopic examination, Table 1), gastrointestinal strongyles (GISs) were the most commonly detected parasites, at 91% of the positive samples (*n* = 235), followed by *Eimeria* spp. (72%), *Moniezia* spp. (38%), and *Nematodirus* spp. (32%) (Table 2). Co-infection with *Eimeria* spp. and GISs was detected in 69% of ruminant samples (*n* = 180), while triple infection involving *Eimeria* spp., GIS, and *Moniezia* spp. was identified in 30% of these samples (*n* = 79). Focusing on the two main parasites detected in ruminant faecal samples (GISs and *Eimeria* spp.), the mean GIS burden across all ruminants was 700 eggs per gram of faeces (EPG). When analysed by species, mean faecal egg counts (FECs) were 632 EPG in goats, 865 EPG in sheep, and 142 EPG in cattle. The mean coccidian burden (*Eimeria* spp.) across all ruminants was 2 670 oocysts per gram of faeces (OPG). Species-specific analysis showed that goats exhibited the highest mean faecal oocyst count (4 729 OPG), which was significantly higher (*p* < 0.001) than those observed in sheep (626 OPG) and cattle (350 OPG). In addition, trematode eggs were detected in 13% and 7% of all ruminant faecal samples for *Dicrocoelium dendriticum* and *Fasciola hepatica*, respectively. *Dicrocoelium dendriticum* eggs were identified in 6% of all goat samples (*n* = 119), 20% of all sheep samples (*n* = 117), and 9% of all cattle samples (*n* = 23). Similarly, *F. hepatica* eggs were detected in 2% of goats, 9% of sheep, and 17% of cattle.

**Table 2:** Infection rate by gastrointestinal parasites detected by coproscopic examination (flotation method, McMaster technique) and mean infection intensity in ruminant faecal samples collected in Corsica (*n* = 259; 119 from goats, 117 from sheep, and 23 from cattle).

Parasites	Positive ruminant samples (%)	Mean parasites per gram of faeces in positive samples [95% CI]
Nematodes		
Eggs of gastrointestinal strongyles (GISs) undifferentiated by coproscopic method (*Haemonchus*, *Ostertagia, Trichostrongylus*, etc.)	235 (91%)	700 [463–939]
Eggs of *Nematodirus* spp.	83 (32%)	69 [54–83]
Eggs of *Trichuris* spp.	68 (26%)	130 [20–50]
Eggs of *Strongyloides* spp.	15 (6%)	102 [51–153]
Lung worms’ larvae (*Dictyocaulus* sp., etc.)	67 (26%)	NI
Cestodes (eggs)		
*Moniezia* spp.	99 (38%)	NI
Trematodes (eggs)		
*Dicrocoelium dendriticum*	33 (13%)	NI
*Fasciola hepatica*	17 (7%)	NI
*Paramphistomum* spp.	0	NI	
Coccidia			
Oocysts of *Eimeria* spp.	186 (72%)	2 670 [1 204–4 137]	
Total endoparasites	246 (95%)	NI	

Abbreviations: NI; no informed data. Due to the low sensitivity of the coproscopic method for these parasites, only the presence or absence of the parasite is informed.

Among the other domestic animal species, which were individually and less frequently sampled during this study, GIS eggs were detected in 9 out of 14 horses (64%). The mean FEC in horses was estimated at 172 EPG. In pigs, 5 out of the 6 sampled individuals were found to be infested. Strongyle eggs were identified in pigs, with a mean FEC of 2 390 EPG. Additionally, coccidian oocysts were detected in 2 pigs, with a mean faecal oocyst count (FOC) of 420 OPG.

### *Cryptosporidium* and *Giardia* spp. identification

Among the 281 faecal samples analysed, *Cryptosporidium* spp. DNA was detected in 11 samples, resulting in a pool-level prevalence of 3.9% (95% CI: 2.0%–6.9%) (Table 3). Of these 11 positive samples, 9 were from goat herds, while one each was detected in a sheep and pig herd (Table 3). Among the 159 ruminant farms sampled, *Cryptosporidium* spp. was detected in at least one sample on 9 farms, yielding an apparent herd-level prevalence of 5.7%. Nine positive samples came from adult females (8 goats and one sheep) and two samples from young goats (< 6 months old) raised on the same farm. Sheep and goats in Corsica are reared under similar semi-extensive farming systems and were sampled following the same protocol implemented by GDS Corse (parasitism monitoring programme, see Materials and methods). However, when comparing these two species of small ruminants, a statistically significant difference (using Pearson’s Chi-squared test) in *Cryptosporidium* spp. pool-level prevalence was observed, with goats (10 positive samples of 121 analysed) showing a higher infection rate than sheep (1/117) (*p* < 0.001). The 11 faecal samples testing positive for *Cryptosporidium* spp. were collected from 10 distinct municipalities, corresponding to 9.8% of the total surveyed area (Fig. 1). Geographically, 10 of the 11 positive samples originated from the Corse-du-Sud department (85 analysed samples), while only one was detected in Haute-Corse (*n* = 196). The pool-level prevalence of *Cryptosporidium* in Corse-du-Sud (11.8%) was significantly higher than in Haute-Corse (0.5%) (*p* < 0.001) (Fig. 1). Using a *t*-test, no statistically significant association was found between the detection of *Cryptosporidium* spp. in a sample and the level of gastrointestinal parasite infection (*p* > 0.1).

**Table 3:** *Cryptosporidium* spp.-positive samples and identified species by host.

Collected animals	Sample number	*Cryptosporidium* spp.-positive (%)	Identified species
Sheep	117	1 (0.8%)	1 *C. parvum*
Goats	121	9 (7.4%)	5 *C. parvum*, 1 *C. ubiquitum*, 3 *Cryptosporidiu*m spp.
Cattle	23	0	–
Horses	14	0	–
Swine	6	1 (16.7%)	1 *C. scrofarum*
Total	281	11 (3.9%)	6 *C. parvum*, 1 *C. ubiquitum*, 1 *C. scrofarum*, 3 *Cryptosporidiu*m spp.

Three *Cryptosporidium* species were identified among the positive samples: *Cryptosporidium parvum* (*n* = 6), reported in both goats (*n* = 5) and sheep (*n* = 1); *Cryptosporidium ubiquitum* (*n* = 1), detected in a goat pool; and *Cryptosporidium scrofarum*, identified in a pig sample analysed individually (Table 3). For three additional *Cryptosporidium*-positive samples, species-level identification could not be achieved. Subtyping analysis was successfully performed for three of the *C. parvum-*positive samples, revealing the following subtypes: IIdA14G1 (GenBank accession number: PV754094) in two samples from goats, and IIdA17G1 (GenBank accession number: PV75409) in another faecal goat sample. The sequence obtained for *C. scrofarum* was also deposited in GenBank under accession number PV765433. The SSU rRNA phylogenetic tree (Fig. 2) confirmed that the *Cryptosporidium* sequences obtained in this study clustered with their respective reference species retrieved from GenBank, supporting the molecular identification and illustrating their evolutionary relationships within the genus.

**Figure 2:**
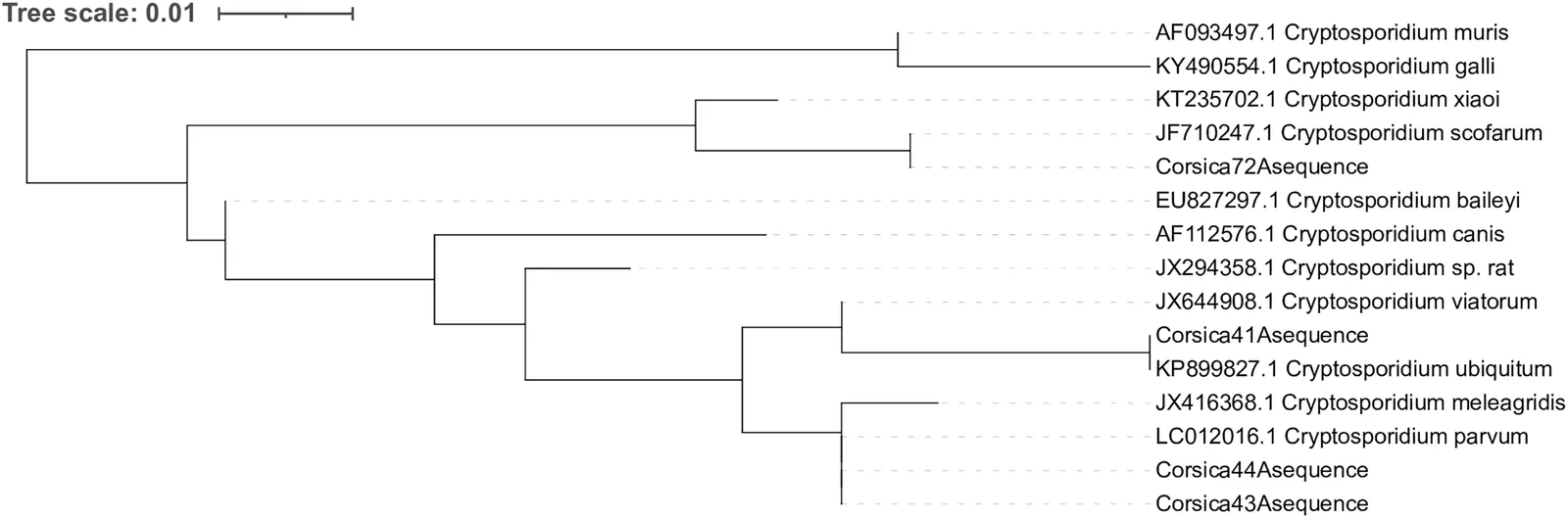
Phylogenetic tree of *Cryptosporidium* species. Samples Corsica 43A, 44A, and 41A correspond to goat faecal samples. Sample Corsica 72A corresponds to a pig faecal sample. The scale bar indicates the number of nucleotide substitutions per site.

*Among the 281 samples analysed for Giardia* spp., *G. duodenalis* assemblage E DNA (GenBank accession number: PV754096) was detected on one goat farm located in Haute-Corse (Fig. 1). A single locus (*gdh*) was successfully sequenced in one positive sample. The apparent pool-level prevalence was 0.4% (95% CI: 0.0%–1.9%) across all sampled animals, and 0.8% (95% CI: 0.02%–4.5%) within the goat population.

## Discussion

### Coproscopic analyses

In the context of pooled samples, most used for this protocol, all results (coproscopic and PCR analyses) should be interpreted as reflecting the infection rate at the sample level. Apparent prevalence could therefore be expected to be lower. However, pooling may also lead to a dilution effect of parasites in the faecal samples, potentially resulting in false negatives.

This study revealed a high pool-level prevalence of internal parasitism in Corsican ruminants, with 93% of the animal samples harbouring at least one parasite. Concerning the ruminant population sampled, GISs were the most common parasites, detected in 91% of faecal samples, followed by coccidia (72%). According to Soulsby *et al.*, [40] and Lambertz *et al.*, [30] FEC values for GINs are classified into the following categories: GIN-free, low (<500 EPG), medium (500–1 000 EPG), and high (> 1 000 EPG). Similarly, faecal oocyst count (FOC) values for *Eimeria* spp. are classified as *Eimeria*-free, low (< 1 800 OPG), medium (1 800–6 000 OPG), and high (> 6 000 OPG) [26, 30]. The mean FEC intensity in Corsican ruminants was 700 EPG, with sheep (865 EPG) and goats (632 EPG) exhibiting medium burden, while cattle (129 EPG) had low burden. Regarding coccidia infection, Corsican ruminants had a mean FOC of 2 700 OPG, with goats (4 749 OPG) falling into the medium range, while sheep (626 OPG) and cattle (350 OPG) had low levels. These findings align with those from studies carried out in Northern Italy [44], where most animals had low to medium GIN burden. However, coccidia infections were more severe, particularly in lambs and kids (20% high infestation).

The Corsican samples were obtained from ruminant herds enrolled in a regional parasitism surveillance program. Participation in such initiatives may indicate increased farmer engagement in animal health management and adherence to biosecurity measures, including the implementation of anthelmintic protocols. However, the GDS Corse protocol does not collect or report detailed data on farm management practices, limiting the extent to which inferences about on-farm interventions can be made. Moreover, as ruminant faecal samples were pooled by five, the actual pool-level prevalence at the individual level may differ. The routine parasitism monitoring protocol used in this study does not include species-level identification of *Eimeria*, GIS, or cestodes. In this context, animal owners are primarily interested in knowing which types of parasites are present and their overall burden, in order to administer appropriate antiparasitic treatments. Therefore, species-level identification of *Eimeria*, GIS, and cestodes was not undertaken, although such information would clearly have been of scientific interest. Molecular tools were used only for species belonging to the genera *Cryptosporidium* and *Giardia*, which were the main focus of this study conducted by the French National Reference Centre for Cryptosporidiosis, Microsporidiosis, and Other Digestive Protozoan Infections (CHU de Rouen, Normandy, France).

The pool-level prevalence of the lancet liver fluke (*Dicrocoelium dendriticum*), *i.e.* 13%, and the large liver fluke (*Fasciola hepatica*), *i.e.* 7%, further highlight the low sensitivity of the McMaster method for detecting trematode eggs. A recent study conducted in Corsica reported the presence of *D. dendriticum* eggs in the bile of 69% of sampled sheep, while nearly 50% of cattle tested seropositive for *F. hepatica* [1]. Regarding *Moniezia* spp., a study conducted on goats in Italy [15] reported an apparent prevalence of 8.4%, which is considerably lower than the 38% observed in the Corsican population across all ruminant species.

### *Cryptosporidium* spp.

Three *Cryptosporidium* species (*C. parvum*, *C. ubiquitum*, and *C. scrofarum*) were identified in domestic animals in Corsica. All three species have been reported to infect humans and are therefore considered zoonotic. To the authors’ knowledge, this is the first study to report the identification of *Cryptosporidium* species in domestic animals on the island. Additionally, a recent survey of wild Corsican rodents reported a *Cryptosporidium* prevalence of 15.4% (*n* = 117), with three species identified: *Cryptosporidium viatorum*, *Cryptosporidium* sp. rat genotype II, and *Cryptosporidium* sp. rat genotype III [19]. No human cases of cryptosporidiosis have been officially reported in Corsica by the Regional Health Agency (*Agence Régionale de Santé*, ARS), and the parasite is not currently monitored in wastewater. Moreover, cryptosporidiosis is not part of routine veterinary diagnostics and has not been the subject of systematic surveillance by research institutions [11]. Consequently, its potential impact on public and animal health remains unclear. Interestingly, *Cryptosporidium* spp. was detected more frequently in Corse-du-Sud than in Haute-Corse (Fig. 1). This geographic variation warrants further investigation and should be discussed with farmers and veterinary stakeholders to identify potential environmental, management, or epidemiological factors that may contribute to this difference.

*Cryptosporidium parvum* was identified in six samples and is recognised as the second most common species causing human cryptosporidiosis worldwide, after *C. hominis*, associated with human infections [36]. In Corsica, the *C. parvum* genotype IId was detected three times in goats from the Corse-du-Sud department, corresponding to two subtypes: IIdA14G1, identified in two municipalities (Sari-d'Orcino and Cuttoli-Corticchiato), and IIdA17G1, detected in one municipality (Tavera). Notably, both IIdA14G1 and IIdA17G1 had never been reported in France prior to this study. Several studies have frequently reported subtype IIdA14G1 in various animal hosts and humans in China [45], as well as in lambs in Spain [35]. Subtype IIdA17G1 has been detected in humans in several European countries, including Finland and England, and in Asia [5, 45]. In addition, it has been reported in various animal species, including young goats in Spain [14]. In mainland France, studies have shown that *C. parvum* genotypes IId and IIa are predominantly detected in sheep from south-western France [3]. Other species, including *Cryptosporidium ubiquitum* and *C. xiaoi*, have also been identified through molecular diagnostics [3]. In another study in the same area specifically targeting goats, *C. xiaoi* was identified as the most prevalent species, while *C. parvum* was only detected in a single case [38]. In Sardinia (Italy), Corsica’s neighbouring island, two *Cryptosporidium* species have been identified in sheep: *C. parvum* (73%) and *C. ubiquitum* (27%). Genotyping further characterised the *C. parvum* isolates as belonging to subtypes IIa15G2R1 and IIdA20G1, and the *C. ubiquitum* isolates to subtype XIIa [13]. *Cryptosporidium scrofarum* (formerly known as *Cryptosporidium* pig genotype II) [28] was identified once on a pig farm in Corse-du-Sud (municipality of Evisa). This represents the first reported identification of this zoonotic species in France. *Cryptosporidium scrofarum*, a species with global distribution [10], has previously been reported in several European countries, including Czechia, Denmark, and Germany. However, within the Mediterranean Basin, its presence has only been documented in wild boars in Spain [32].

Animal transfers between Corsica and mainland Europe are highly limited or virtually non-existent. This is primarily due to the predominance of local breeds (cattle, sheep, goats, and pigs) that are well-adapted to the island’s mountainous environment, as well as the historical presence of certain animal diseases, which significantly constrain animal movements. Notable examples include specific serotypes of the bluetongue virus (BTV) circulating in ruminants [22] and the enzootic presence of Aujeszky’s disease virus in pigs [9]. Consequently, despite the limited movement of livestock between Corsica and mainland Europe, the introduction of zoonotic *Cryptosporidium* species and subtypes through human activities cannot be excluded and should be considered when interpreting the epidemiology of these parasites on the island.

The phylogenetic analyses further supported the molecular identification obtained by sequencing, with the SSU rRNA sequences generated in this study clustering with reference sequences of *Cryptosporidium parvum*, *C. ubiquitum*, and *C. scrofarum*. This clustering confirms the taxonomic placement of the Corsican isolates and demonstrates their close evolutionary relationship with isolates previously reported from livestock and, for *C. parvum* and *C. ubiquitum*, from human infections worldwide. Although the SSU rRNA marker is primarily intended for species-level identification rather than transmission analysis, these results strengthen the evidence that zoonotic *Cryptosporidium* species circulate in Corsican livestock.

This study revealed an overall *Cryptosporidium* spp. pool-level prevalence of 3.9% among all analysed animal samples, with species-specific rates of 7.4% in goat samples and 16.7% in pigs. Based on a single positive result from six sampled animals, the apparent porcine prevalence requires cautious interpretation due to the small sample size. Compared to studies conducted in continental Europe, the prevalence rates observed in Corsica appear to be significantly lower. In south-western France, molecular analyses detected *Cryptosporidium* spp. in 35% of sheep [3] and in approximately 50% of young goats [38]. Similarly, a longitudinal study conducted in Brittany reported an overall prevalence of 70.4% in calves, with farm-level prevalence ranging from 10% to 100% [18].

Importantly, this study used molecular analysis as the sole detection method for *Cryptosporidium* spp., in contrast to many previous epidemiological studies that combined molecular and microscopic techniques, an approach known to yield higher prevalence rates. This methodological difference is exemplified by research from Sardinia, where microscopic examination detected oocysts in over 10% of sheep samples, while concurrent PCR analysis confirmed just 1.6% positivity (15/915 samples) [13]. Several additional factors may have influenced the sensitivity of our detection method: (i) sample processing: using pooled faecal samples likely caused a dilution effect, potentially compromising DNA detection thresholds; and (ii) population characteristics: most samples originated from herds enrolled in a parasite monitoring program, where enhanced biosecurity measures possibly reduced parasite loads below detection limits.

A limitation of this study is that three *Cryptosporidium*-positive samples could not be identified to the species level. Although PCR amplification was successful, sequencing produced unreadable electropherograms, most likely due to mixed *Cryptosporidium* infections within the same sample. The simultaneous presence of multiple species may lead to co-amplification of different genetic templates, resulting in superimposed chromatograms that prevented unambiguous species identification.

### *Giardia* spp.

To date, no human cases of giardiasis have officially been reported in Corsica by the Regional Health Agency, a situation similar to that with cryptosporidiosis (see above). Giardiasis is commonly acknowledged among local veterinary practitioners as a significant cause of diarrhoea in young domestic carnivores, although no formal publication currently reports these data in Corsica. Detection is typically performed using a rapid immunoassay test targeting faecal antigens. A systematic review estimated the worldwide prevalence of dogs and cats to be 15.2% and 12%, respectively [4]. In this Corsican study, *G. duodenalis* Assemblage E was detected in one sample from a goat farm in Haute-Corse (Fig. 1). The pooling protocol used in this study may have reduced the detection rate of positive samples; nonetheless, this single detection represents the first official report of *G. duodenalis* on this island. Assemblage E is not zoonotic and is generally considered ruminant-specific, although it has also been reported in other ungulate species. Further investigations are needed to assess *Giardia* spp. circulation in Corsica in order to better evaluate potential zoonotic reservoirs, particularly among domestic carnivores. This is especially relevant because the zoonotic *G. duodenalis* Assemblages (A and B) infect multiple animal species [25]. A study conducted in France in 2005 using a fluorescent monoclonal antibody test found *G. duodenalis* cysts on 100% of surveyed goat farms in western France, with within-farm prevalence ranging from 10% to 80% of animals [7]. In France, published data are lacking on the *G. duodenalis* assemblages circulating in small ruminant populations. However, a European study conducted in calves reported that *G. intestinalis* Assemblage E was predominant in most European countries, whereas in France it ranked second (39% of positive samples), after Assemblage A (61%) [20]. *Giardia intestinalis* Assemblage E has frequently been reported in other Mediterranean countries, particularly in Spain, where it has been identified in cattle, goats, pigs, and wild boars, as well as in Italy, in both cattle and wild ruminants [2].

## Conclusions

This study demonstrated that gastrointestinal parasites are widespread in Corsican livestock, with GINs and *Eimeria* spp. being the predominant parasites detected. Overall parasite burden was generally low to moderate. Further studies are needed to evaluate the efficacy of current parasite control strategies and to investigate the possible emergence of anthelmintic resistance in the semi-extensive farming systems practised in Corsica.

Consistent with previous findings from mainland France and the Mediterranean region [2, 7, 11, 13, 37], this study confirmed the presence of *Cryptosporidium* spp. and *G. duodenalis* in Corsican livestock. To the authors’ knowledge, this is the first documented evidence of these protozoan parasites in domestic animals on the island. These findings are particularly significant for local veterinarians and farmers, as neither parasite is currently included in routine veterinary diagnostics for livestock. Future studies based on individual sampling of young diarrhoeic ruminants (sheep, goats, and cattle) would provide a more accurate assessment of the clinical impact of *Giardia* and *Cryptosporidium* infections on Corsican livestock. Such data would help to inform local stakeholders about *Giardia* and *Cryptosporidium* infections in these animals on the island.

Lastly, the identification of three zoonotic *Cryptosporidium* species demonstrates that Corsican livestock may constitute a reservoir of parasites of public health importance. Although no human cases have officially been reported on the island to date, the circulation of *C. parvum*, *C. ubiquitum*, and *C. scrofarum* highlights the need for integrated surveillance under a One Health framework. Future studies should investigate the prevalence and genetic diversity of *Cryptosporidium* spp. and *G. duodenalis* in humans, particularly in areas where zoonotic *Cryptosporidium* species were detected. Notably, this study also reports the first identification of *C. scrofarum* in France, extending the known geographical distribution of this zoonotic species.
